# Flanker interference at both stimulus and response levels decreases with age

**DOI:** 10.1007/s00221-023-06773-9

**Published:** 2024-02-02

**Authors:** Nunzia Valentina Di Chiaro, Nicholas Paul Holmes

**Affiliations:** 1 School of Psychology, University of Nottingham, University Park, Nottingham, UK; 2 School of Sport, Exercise and Rehabilitation Sciences, University of Birmingham, Birmingham, UK

**Keywords:** Vision, Attention, Cognitive control, Perceptual interference, Response interference, Ageing

## Abstract

When trying to identify the colour of a target, people’s performance is impaired by nearby distractors of different colours. It is controversial whether these interference effects originate from competing stimuli, competing responses or from both simultaneously. These interference effects may also differ depending on a person’s age. Comparisons between studies show mixed results, while differences in experimental design and data analysis complicate the interpretation. In our study, we manipulated the relative proportions of congruent and incongruent trials with respect to both stimuli and responses. Considering this aspect, we asked whether people resolve stimulus and response interference differently at different ages. 92 children (6–14 years), 25 young adults (20–43 years) and 33 older adults (60–84 years) performed a coloured version of the Eriksen flanker task. Since reaction times and errors were correlated, inverse efficiency scores were used to address speed-accuracy trade-offs between groups. Absolute interference effects were used to measure relationships with age. The results showed first, unexpectedly, that response interference was comparable between stimulus- and response-balanced conditions. Second, performance at all ages was significantly influenced both by competing stimuli and responses. Most importantly, the size of interference effects decreased with age. These findings cast some doubt on the conclusions of previous studies, and raise further questions about how cognitive control is best measured across the lifespan.

**Supplementary Information:**

The online version contains supplementary material available at 10.1007/s00221-023-06773-9.

## Introduction

Interference control is the ability to ignore interfering information to maintain attention on a task. It represents an essential skill in everyday life, to avoid us being distracted by irrelevant information (Diamond 2013).

Irrelevant information can interfere at any or all phases of task processing, for example during stimulus processing, response selection and/or response execution. Accordingly, interference control has been studied at the level of stimulus identification and target detection, as well as at the response level. Different and non-overlapping attentional mechanisms in the brain have been found for resolving these forms of cognitive interference (van Veen et al. 2001; van Veen and Carter 2005).

Different researchers have discussed whether irrelevant information may interfere differently depending on a person’s age. At any age, behaviour can be influenced by interference from irrelevant information, however the size of interference at both stimulus and response levels has been argued to differ with age. For example, children and older adults are reported to be less able to overcome distraction from irrelevant information than young adults (Vu and Proctor 2008; Li et al. 2009). Several studies have aimed to understand how people at different ages resolve interference originating from competing stimuli and responses (Jongen and Jonkman 2008; Killikelly and Szucs 2013; Cragg 2016; Hirst et al. 2019).

Jongen and colleagues (Jongen and Jonkman 2008) recruited three groups of children aged 6–7, 8–9, and 10–12 years, and a group of young adults aged 18–28 years. They administered a colour object Stroop task in which participants had to identify, by pressing a button as quickly as possible, the printed colour of familiar objects presented in their usual or an unusual colour (e.g., a red strawberry vs. a blue strawberry). Stimulus and response interference were manipulated by the colour of the stimulus and the mapping of these colours to the response buttons. In the stimulus interference condition, the unusually-coloured stimuli were mapped onto the same response button as the object’s usual colour. In the response incongruent condition, the unusual colour was mapped onto a different response button than the object’s usual colour. The results suggested that interference at the stimulus level—the slowing of reaction times for stimulus incongruent compared to fully congruent stimuli—was already developed at 6–7 years old, because no differences in the size of the stimulus interference effect were found between the age groups. At the same time, the response interference effect was equally strong in all age groups, however, children were less accurate than adults.

According to more recent studies (Cragg 2016; Hirst et al. 2019), control over competing stimuli has not fully developed by 7 years of age. Cragg (2016) tested two groups of children aged 7 and 10 years old, and a group of young adults of mean age 21 years, using a colour version of the Eriksen flanker task (Eriksen and Eriksen 1974). Participants were asked to indicate the colour of a central line while ignoring the colours of two flanking parallel lines. Both groups of children experienced greater stimulus interference than the young adults, and 7 year old children showed more stimulus interference (resulting in longer reaction times) than the 10 year olds. Regarding response interference, there were no differences in the amount of interference experienced across age groups. Interestingly, Hirst et al. (2019) observed in children between the ages of 6 and 11 years, that stimulus interference facilitated the correct response, while response interference facilitated the incorrect response. Comparing these two patterns across the response time distribution, the authors showed that response interference occurred at the longest response time latencies, while stimulus interference remained constant.

To our knowledge, few studies have investigated how people resolve the interference originating from competing stimuli and responses in later life. Hirst et al. (2019) suggested that stimulus interference control could be more susceptible to age-related changes than response interference, finding that adults aged 61–85 years showed slower responses and greater interference in the stimulus- than the response-interference condition. No specific age-related patterns in the response time distribution, as in childhood, were found. In another study, Killikelly and Szucs (2013) showed that both stimulus and response competition contributed to the overall interference effect, however the interference was comparable among age groups. They tested adolescents (16–17 years old), young adults (23–30 years old), and middle-age adults (45–62 years old).

Looking across the reported studies, it is difficult to answer the question about how people at different ages resolve stimulus and response interference, because differences in experimental design and data analysis between studies complicate such comparisons.

In the reported studies, different measures have been used to calculate interference effects. Some studies used the ratios (relative scores) of incongruent: congruent responses as an interference measure (Cragg 2016; Hirst et al. 2019), whereas other studies used differences (absolute scores) (Jongen and Jonkman 2008; Killikelly and Szucs 2013). In light of these considerations, it may be important to use the same measure of interference, making the comparison among studies easier. In the original version of this manuscript, we tried to test whether the ratio or absolute scores were better measure of flanker interference effect, however we found no significant results to conclude that one measure was better than the other (see supplementary material—Sect. 3).

The proportions of congruent and incongruent trials also influence the size of interference effects (Cragg 2016). As reported by Hasshim and colleagues (Hasshim and Parris 2014), the associations between stimuli and responses are learnt during an experiment, and participants may use them to predict or prepare for upcoming stimuli, which may result in more accurate or faster responses, rather than interfering on the majority of trials. In the reported studies, the proportion of trials for each condition influenced the congruence in relation to stimuli and responses (see Fig. S1 in supplementary material—Sect. 1). In Cragg (2016), Hirst et al. (2019) and Jongen et al. (2008), the proportion of trials for each condition was equal. Looking at the stimulus level (the colour of target and distractors), 33.3% trials were congruent and 66.7% trials were incongruent, resulting in a stimulus imbalance. Looking at the response level (the response associated with the target and distractors), 66.7% trials were congruent and 33.3% were incongruent, resulting in a response imbalance at the same time. In Killikelly and Szucs (2013), the number of response incongruent trials was doubled in comparison to the other conditions, and this doubling gave an equal proportion of congruent and incongruent trials at the response level (50 and 50%), and an unequal proportion of congruent and incongruent trials at the stimulus level (25 and 75%, respectively). In these experiments, researchers must decide whether to balance the proportion of trials with respect to the stimuli or to the responses.

To our knowledge, no previous study has taken into account these aspects in the experimental design and data analysis, which may affect the interpretation of results, and complicate between-study comparisons.

Our first aim was to compare the interference effects in stimulus- and response-balanced conditions, in which there were different proportions of stimulus- and response-congruent trials. In the stimulus-balanced (response-imbalanced) condition, the number of congruent trials was doubled in order to balance at the stimulus level, so 75% of trials were response-compatible and 25% response-incompatible. In the response-balanced condition (stimulus-imbalanced), the number of stimulus–response incongruent trials was double that of the other conditions in order to balance at response level, so 50% of trials were response-compatible and 50% response-incompatible. Increasing the proportion of stimulus–response incongruent trials decreases the interference effect (Schmidt and Besner 2008). We expected to find greater response interference effects in the stimulus-balanced condition because the distractors of the stimulus incongruent trials—associated with the congruent response—facilitate more accurate and faster responses, increasing the difference between the stimulus and stimulus–response incongruent trials. In response-balanced conditions, there is the same probability that the distractors are associated with a congruent or incongruent response, which should prevent any facilitation of the response.

Taking into account these methodological factors, the main aim was then to assess how stimulus, response and general interference effects differ across three age groups, from 6–14, 20–43, and 60–84 years.

## Method

### Participants

We recruited 150 participants in three age groups: 92 children (6–14 years old, mean ± SD = 8.8 ± 2.1 years, 47 females, 83 right-handed by self-report), 25 young adults (20–43 years old, mean ± SD = 28.2 ± 5.1 years, 16 females, 21 right-handed and 1 ambidextrous by self-report) and 33 older adults (60–84 years old, mean ± SD = 70.2 ± 6.5 years, 19 females, 19 right-handed, 4 left-handed, 1 ambidextrous, 9 not reported). We recruited child and older-adult participants during two outreach events, the duration of which was set a priori, resulting mostly in convenience sampling; no a priori power analysis was performed to justify the sizes of either group. For the young adults, we relied on our prior knowledge that interference effects produce large effect sizes. For this reason, recruiting 24 participants was already known to be a large sample size for this group.

Young adults were students and staff at the University of Nottingham. They were recruited via posters and email advertisements to a mailing list of participants who had previously participated in other experiments. Children, and one third of the older adults, were recruited via public engagement events at the University: children via ‘Summer Scientist Week’ (July 2019) and older adults via ‘Silver Scientist Day’ (June 2019) and a ‘Wellbeing Age Conference’ (October 2019). The remaining older adults were recruited via the University’s volunteer database and word of mouth. Children received tokens to be spent on games at the event, and older adults who participated received a university gadget. Young adults and some older adults received an inconvenience allowance of £5.

All participants were fluent in English and reported no history of neurological or psychiatric disorders. All had normal or corrected to normal vision. Older adults tested in the laboratory were briefly examined with a ‘Snellen chart’ (Snellen 1862) to test visual acuity (mean ± SE = 7.57 ± 0.76). The Montreal Cognitive Assessment (MoCA) was used to test cognitive functioning (mean ± SE = 25.97 ± 2.50). The older adults understood the task instructions, and all performed the task better than chance (binomial test, *p* ≤ 0.05). The correlations between the MOCA score and the perceptual, response and general interference effect are shown in the supplementary material—Sect. 2. One participant did not perform the MoCA test due to a lack of time during the public event.

Written informed consent was obtained from each participant, and the children’s carer before inclusion in the study. The study received ethical approval from the research ethics committee of the University of Nottingham (reference: SoPEC1196).

### Stimuli and apparatus

Stimuli were coloured squares on a black background. The RGB codes of each colour were: red = [255 0 0], blue = [0 0 255], green = [0 255 0], yellow = [255 255 0]. The sides of each square were 2.5° from a viewing distance of 57 cm, and there was 1.3° edge-to-edge separation between adjacent squares.

Stimuli were presented via a desktop Lenovo PC running Windows7 on a monitor 16″ (resolution 1920 × 1080) for participants tested in the laboratory, and via a HP ProBook 430 G2 running Windows 7 on a monitor 15.4″ (resolution 1366 × 768) for participants recruited via the public engagement events. Responses were collected with a standard high-speed USB2 computer keyboard. Stimulus presentation and response collection were controlled by custom scripts written using MATLAB and PsychToolBox 3 libraries (Brainard 1997). IBM SPSS Statistics 21.0 was used for statistical analysis. All code and raw data are freely available at https://osf.io/sx3je/.

### Task description

A coloured version of the Eriksen flanker task was administered. Two flanker squares of the same colour (red, blue, green or yellow) were presented to the left and right of, and began 200 ms earlier than, a middle square that was the target. In the *congruent condition* (C), the target and flankers were the same colour. In the *stimulus incongruent* (SI) condition the target and flankers differed in colour but were both mapped to the same response hand and button. In the *stimulus–response incongruent* (SRI) condition, the target and flanker colours were different and were mapped to different response hands and buttons, evoking both stimulus and response interference at the same time. Participants were instructed to respond to the colour of the middle square (the target) using the left and right index fingers, and to ignore the surrounding squares (the flankers). They were encouraged to respond as quickly and as accurately as possible.

The colours were associated with two response buttons on a standard QWERTY keyboard; two colours were associated with a left key and left index finger response (button ‘c’) and two colours were associated with a right key and right index finger response (button ‘m’). The four colours were combined in three response assignments (red/blue and green/yellow; red/green and blue/yellow; red/yellow and blue/green) that were counterbalanced across participants. Coloured square labels of the target colours assigned to each key response were placed above the relevant key during the entire experiment to act as a reminder to participants.

### Procedure

The participants were sitting comfortably in a quiet and dimly lit room, with their chin on a head-and-chin rest at a viewing distance of 57 cm from the monitor. Children did not use the head-and-chin rest because it was reported as uncomfortable by the first few children. Before starting the experiment, each participant was asked to name the colour of four coloured squares (red, blue, green and yellow) shown on the screen, to check for gross colour-blindness.

**Fig. 1:**
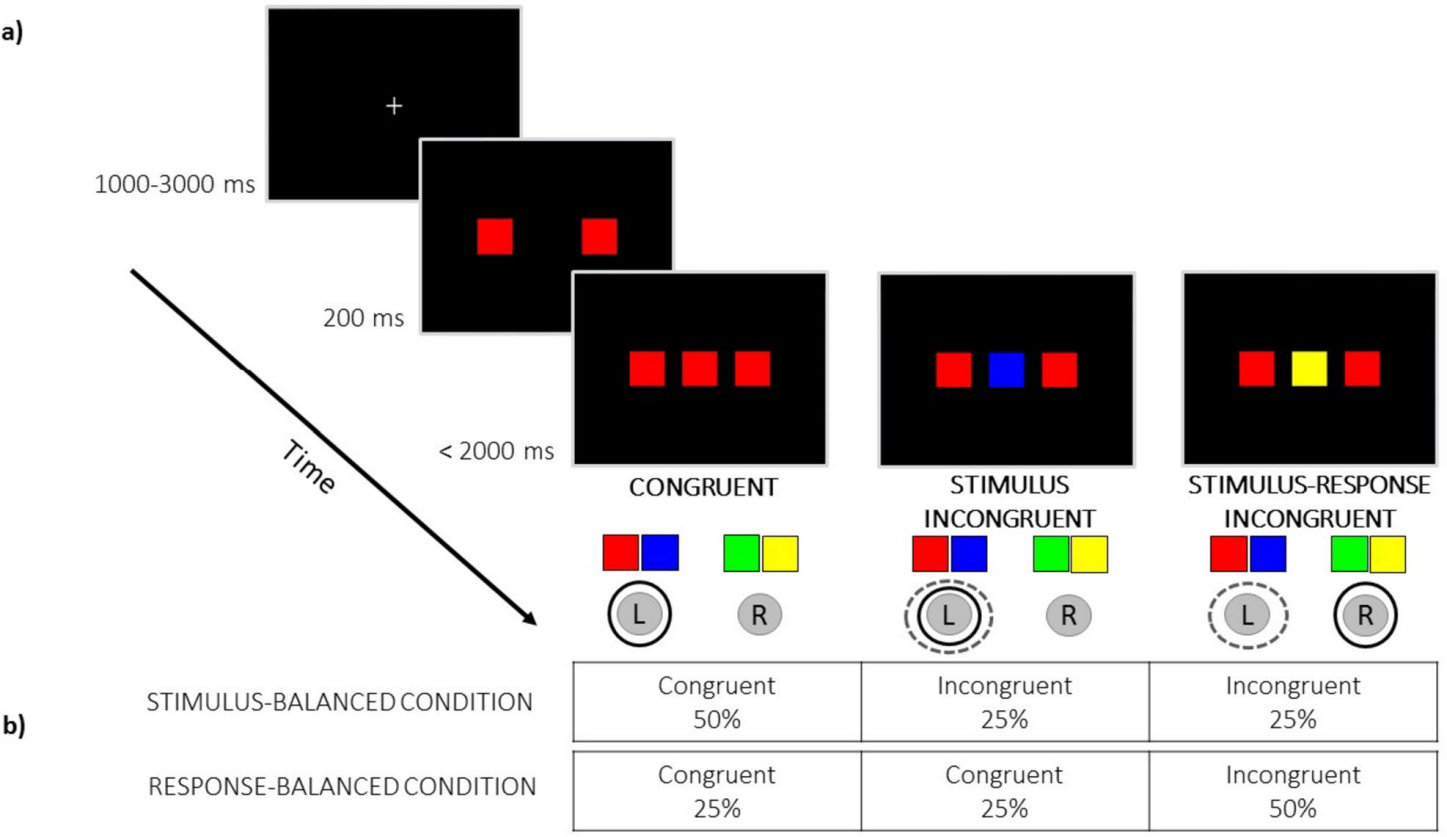
**a** The illustration of trial sequence in the colour version of Eriksen flanker task. The stimuli are not to scale. For each condition, correct response has been *circled with a solid black line* and the incorrect response in a *broken grey line*. *L* left response button, *R* right response button. **b** The proportion of congruent trials in the stimulus-balanced and response-balanced conditions in our study.

As shown in Fig. 1a, each trial started with the presentation of a fixation cross in the middle of the screen for a pseudorandom interval of uniform distribution 1000–3000 ms. Two flanker squares were presented, then 200 ms later, the target was presented between the two flankers until the participant’s response or until a maximum of 2000 ms, before the stimuli were removed.

Children went through one practice block of 16 pseudorandomised trials. The practice blocks were counterbalanced between stimulus-balanced and response-balanced conditions between children. Young and older adults went through two practice blocks of 32 pseudorandomised trials each. The practice blocks were counterbalanced between stimulus-balanced and response-balanced conditions. For the young adults, if accuracy in the practice blocks was lower than 75% and/or the mean RT was longer than 500 ms, feedback was shown to encourage them to improve accuracy and/or speed in the next block. In this case, each practice block was repeated up to three times, before the participant started the experiment. For children and older adults, feedback was shown when accuracy was lower than 75% and/or RT longer than 900 and 700 ms respectively. In this case, the blocks were not repeated, due to the lack of time during events, and the participants then went through the experiment.

During the experiment, stimulus-balanced and response-balanced conditions were blocked and presented in counterbalanced order across participants. In stimulus-balanced (response-imbalanced) blocks, the proportion of congruent trials was double that in the other conditions (50% C, 25% SI, 25% SRI) whereas in response-balanced (stimulus-imbalanced) blocks, the proportion of SRI trials was double that in the other conditions (25% C, 25% SI, 50% SRI) (Fig. 1b). Children performed one stimulus-balanced block (16 C, 8 SI, 8 SRI trials) and one response-balanced block (8 C, 8 SI, 16 SRI trials), giving 64 trials in total. Young adults went through two stimulus-balanced blocks (48 C, 24 SI and 24 SRI trials) and two response-balanced blocks (24 C, 24 SI and 48 SRI trials), giving 384 trials in total. Older adults went through one stimulus-balanced block (32 C, 16 SI, 16 SRI trials) and one response-balanced block (16 C, 16 SI, 32 SRI trials), giving 128 trials in total.

### Statistical analysis

Eleven children were excluded from the analysis. During the public engagement event, every child was allowed to take part in the study, but the data were not considered for analysis when the children were younger than six years old, as performance was very poor. Seven children were excluded for this reason. Two children were excluded because their careers reported diagnoses of developmental disorders, and two due to technical problems during the experiment. A final sample of 81 children were considered for analysis. One young adult was excluded because they were unable to complete the study, therefore a sample of 24 young adults were considered for analysis. Two older adults were excluded: one reported a diagnosed colour blindness, and one was unable to complete the study. A sample of 31 older adults were included in the analysis. Using binomial tests, all participants performed better than chance at the *p* ≤ 0.05 level: children were correct on at least 38 out 64 trials, young adults on at least 208 out 384 trials, and older adults on at least 73 out 128 trials.

A log-10 transformation of the variable age was performed in order to address the skewed distribution when looking at age as a continuous predictor. Percentage of correct responses and mean RTs for correct responses were calculated. These measures were combined into the inverse efficiency score (IES) (see supplementary material—Sect. 2). IES consists of RTs divided by the proportion of correct responses (Townsend and Ashby 1978; Bruyer and Brysbaert 2011). Greater values of IES indicate worse performance. By calculating IES before the measure of interference effects, we dealt with the potential problems of speed-accuracy trade-offs, and reduced two performance variables down to one—simplifying our analysis. The importance of using combined scores of accuracy and speed to compare the performance among different ages has been supported before (Salthouse 2010). The mean percentage of correct responses, RTs and IES were calculated for C, SI and SRI conditions for each participant within and between blocks. The descriptive statistics for the three groups are reported in Table 1.

**Table 1:** Mean (M) and standard deviation (SD) of proportion of correct responses (accuracy), reaction times (RT) and inverse efficiency scores (IES), for each age group, under total, congruent, stimulus incongruent and stimulus–response incongruent condition across blocks

	Total		Congruent		Stimulus Incongruent		Stimulus response incongruent	
	*M*	(SD)	*M*	(SD)	*M*	(SD)	*M*	(SD)
Children (*n* = 81)								
Accuracy (%)	94.1	(6.14)	95.6	(5.84)	95.7	(7.03)	91.4	(9.40)
RT (ms)	861	(181)	823	(172)	853	(197)	913	(193)
IES (ms)	928	(260)	870	(217)	911	(287)	1028	(337)
Young adults (*n* = 24)								
Accuracy (%)	92.6	(4.92)	94.5	(4.39)	94.2	(4.52)	88.9	(7.63)
RT (ms)	456	(66.2)	427	(69.6)	457	(69.0)	492	(66.4)
IES (ms)	493	(72.5)	454	(76.8)	487	(74.5)	558	(84.2)
Older adults (*n* = 31)								
Accuracy (%)	97.2	(4.30)	97.1	(4.75)	98.1	(3.76)	96.8	(5.28)
RT (ms)	630	(114)	616	(120)	631	(113)	649	(116)
IES (ms)	654	(132)	640	(137)	647	(122)	677	(141)

Ratio and absolute scores between interference conditions were calculated to isolate the three interference effects using IES (De Houwer 2003). We looked at all the interference measures, but only absolutes scores were used to test the main experimental hypotheses based on the results reported in supplementary material—Sect. 3. Respectively, the ratio and absolute scores of the perceptual interference effect were calculated by dividing or subtracting the SI and C conditions (SI/C or SI–C); the response interference effect was calculated by dividing or subtracting the SRI and SI conditions (SRI/SI or SRI–SI). Lastly, a general interference effect was calculated by dividing or subtracting the SRI and C condition (SRI/C or SRI–C). Ratio scores greater than 1, and absolute scores greater than 0 indicate interference effects. These scores were calculated within and between blocks. A bootstrap resampling technique was used to investigate whether ratio or absolute scores were better—in terms of their distributions—as a parametric measure of the interference effect. Looking at the Q-Q plot of the original raw data, we found a larger deviation from normality at the upper tail for general interference effects measured using ratio scores in comparison to absolute scores. As reported in the supplementary material—Sect. 3, we did not reach a strong conclusion whether ratio or absolute scores were better measure of interference effect. For only reason to simplify the number of the statistical tests, we arbitrarily chose the absolute scores based on their marginally more normal distributions.

Following a reviewer’s request, we re-analysed the data excluding one outlier belonging to the children group (see supplementary material—Sect. 5). Statistical significance was assessed with *α* = 0.05. Degrees of freedom and p values were corrected using Greenhouse–Geisser estimates of sphericity where the assumption was violated. The partial eta-squared were calculated as an estimate of the effect size (Lakens 2013).

## Results

### Stimulus-balanced versus response-balanced

A repeated measures ANOVA with condition (s*timulus-balanced versus response-balanced*) as within-subject factor and log10(age) as a covariate was performed separately for perceptual, response and general interference effects (separate analyses are required because both perceptual and response interference effects share variance from the SI condition).

**Fig. 2:**
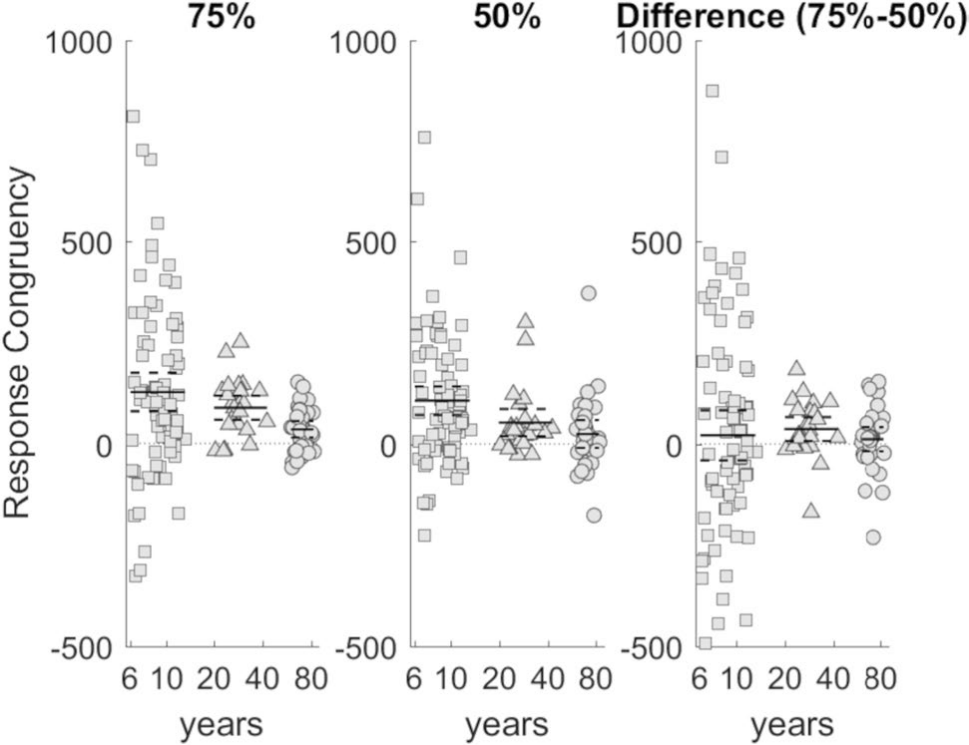
Response interference effect (IES, ms) in stimulus-balanced (75% response-congruency) and response-balanced (50% response-congruency) condition. Mean and 95% confidence intervals reported for each age group

For perceptual, response and general interference, there was no significant effect of condition [*F*(1,134) = 0.682, *p* = 0.410, *ηp*^2^ = 0.00506, MSE = 15,138; *F*(1,134) = 0.087, *p* = 0.769, *ηp*^2^ = 0.000649, MSE = 24,646; *F*(1,134) = 0.863 *p* = 0.355, *ηp*^2^ = 0.00640, MSE = 25,320 respectively]. Although the differences were in the predicted direction, interference effects were comparable in stimulus-balanced and response-balanced conditions (Table 2). There was no significant interaction between condition and age for perceptual, response and general interference effects [*F*(1,134) = 0.159, *p* = 0.691, *ηp*^2^ = 0.00119, MSE = 15,138; *F*(1,134) = 0.002, *p* = 0.965, *ηp*^2^ = 0.0000149 MSE = 24,646; *F*(1,134) = 0.070, *p* = 0.792, *ηp*^2^ = 0.000522, MSE = 25,320, respectively] (Fig. 2).

**Table 2:** Mean (M), standard deviation (SD), effect size (Cohen’s d) of interference effects (IES, ms) in stimulus and response-balanced conditions

	Stimulus-balanced *(75% response-congruency)*			Response-balanced *(50% response-congruency)*		
	*M*	SD	*d*	*M*	SD	*d*
Perceptual interference	42.9	118	0.364	20.4	150	0.136
Response interference	100	174	0.575	78.6	139	0.565
General interference	143	179	0.799	98.9	215	0.460

### Perceptual, response and general interference effects across age groups

A univariate ANOVA was performed, submitting the perceptual, response and general interference effects as dependent variables and log10(age) as a covariate. Significant effects of age were planned to be followed-up with independent samples *t* tests to compare interference effects between the age groups.

**Fig. 3:**
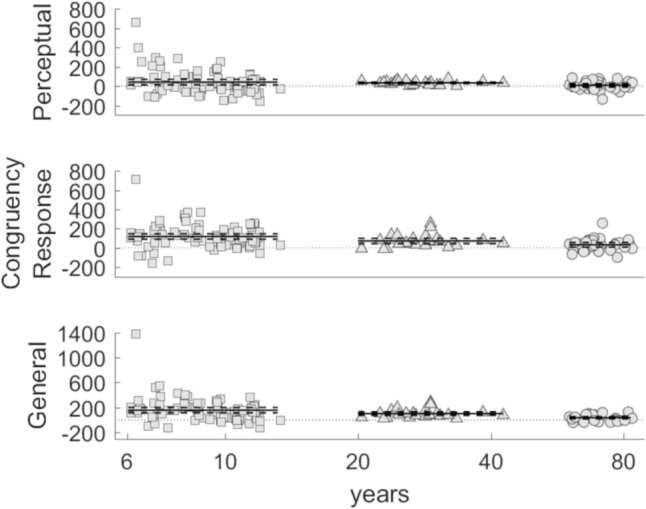
Perceptual, response and general interference effects (IES, ms) decrease across age groups: children (*squares*), young adults (*triangles*) and older adults (*circles*). Mean and 95% confidence interval are shown for each age group

For the perceptual interference effect, a significant effect of age was found, *F*(1,134) = 5.31, *p* = 0.023, *ηp*^2^ = 0.0381, MSE = 10,297. A subsequent analysis between groups showed that children and young adults experienced more perceptual interference than older adults. Children and young adults showed comparable perceptual interference. For the response interference effect, a significant effect of age, *F*(1,134) = 16.9, *p* < 0.001, *ηp*^2^ = 0.112, MSE = 11,317, found that children showed more response interference compared with both young adults and older adults, and young adults showed more response interference than older adults. For the general interference effect, a significant effect of age, *F*(1,134) = 19.2, *p* < 0.001, *ηp*^2^ = 0.125, MSE = 23,483 showed that children had greater general interference compared to young adults and older adults. Young adults showed more interference than older adults (Table 3). As illustrated in Fig. 3, younger people showed greater perceptual, response and general interference compared to older people.

**Table 3:** Interference effects (IES, ms) in age groups. Mean (*M*), standard deviation (SD), effect size (Cohen’s d), *t *test (*t*), degree of freedom (*df*)

	Children (*N* = 81)			Young adults (*N* = 24)			Older adults (*N* = 31)		
	*M*	SD	*d*	*M*	SD	*d*	*M*	SD	*d*
Perceptual	40.8	129	0.316	32.7	19.9	1.64	6.84	47.9	0.143
Response	118	127	0.929	71.1	66.5	1.07	30.3	65.3	0.464
General	158	196	0.806	104	67.7	1.54	37.2	47.3	0.786

	Children vs. Young adults				Children vs. Older adults				Young vs. Older adults			
	*t*	*df*	*d*	*p*	*t*	*df*	*d*	*p*	*t*	*df*	*d*	*p*
Perceptual	0.540	91.3	0.053	0.591	2.03	110	0.192	0.045	2.72	42.2	0.367	0.009
Response	2.37	74.7	0.231	0.020	4.75	101	0.449	<0.001	2.28	53	0.307	0.027
General	2.11	101	0.206	0.037	5.18	99.9	0.489	<0.001	4.30	53	0.580	<0.001

### *Comparing the current data with *Cragg (2016)* and *Hirst et al. (2019)

We asked Cragg (2016) and Hirst et al. (2019) if we could reanalyse the raw data of their studies to clarify the differences between studies. We calculated IES and the absolute interference effects. We calculated Cohen’s d to allow comparison among studies. Summary data are available in Table S1 in supplementary material—Sect. 4. Our findings were generally consistent with these previous studies and across age groups, however both the absolute (ms) and statistical (Cohen’s d) effect sizes varied substantially, making definitive conclusions hard to draw.

### Results using the same number of trials across groups

**Fig. 4:**
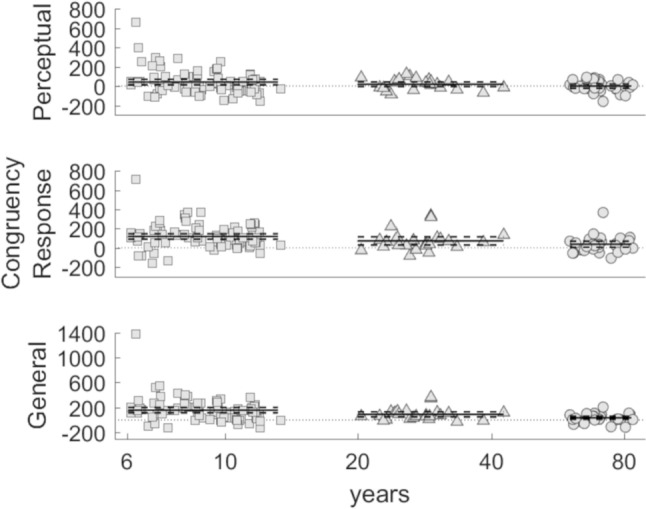
Using the same number of trials, perceptual, response and general interference effects decrease across age groups: children (*squares*), young (*triangles*) and older adults (*circles*) Mean and 95% confidence interval are shown for each age group

In response to a reviewer of an earlier version of this work, we also analysed the data using only the first 32 trials of the stimulus-balanced block (the first 16 C, 8 SI and 8 SRI trials) and the first 32 trials of the response-balanced block (the first 8 C, 8 SI and 16 SRI trials) for both young and older adults, so that all three age groups had the same number of trials. All trials of each condition were used for children. The results were the same. The ratio and absolute scores of the general interference effect were significantly correlated [*r*(134) = 0.863, *p* < 0.001]. Resampled ratio and absolute scores were similarly normally distributed, again the Q-Q plot revealed a larger deviation from normality at the upper tail for general interference effects measured using ratio scores in comparison to absolute scores. There was no significant effect of condition (s*timulus-balanced versus response-balanced*) for perceptual, response and general interference [*F*(1,134) = 0.693, *p* = 0.407, *ηp*^2^ = 0.00515, MSE = 16,817; *F*(1,134) = 0.418, *p* = 0.519, *ηp*^2^ = 0.00311, MSE = 27,466; *F*(1,134) = 1.66 *p* = 0.200, *ηp*^2^ = 0.0122, MSE = 27,863 respectively] (see Table S4 in supplementary material—Sect. 6). For perceptual, response and general interference effect, a significant effect of age was found [*F*(1,134) = 7.52, *p* = 0.007, *ηp*^2^ = 0.0531, MSE = 10,930; *F*(1,134) = 12.7, *p* = 0.001, *ηp*^2^ = 0.0866, MSE = 13,024; *F*(1,134) = 19.4, *p* < 0.001, *ηp*^2^ = 0.127, MSE = 24,743 respectively] (see Table S5 in supplementary material—Sect. 6). In summary, the interference effects decreased significantly with age when a reduced number of trials were considered (Fig. 4).

## General discussion

In our study, we examined how children, younger and older adults resolve interference originating from irrelevant stimuli, competing responses, and from both competing stimuli and responses at the same time. Looking across the literature (Jongen and Jonkman 2008; Killikelly and Szucs 2013; Cragg 2016; Hirst et al. 2019), it was difficult to answer this question, because different measures of interference have been used in different studies. Furthermore, previous studies did not all take into account the observation that the proportions of stimulus–response congruence may influence the size of the response interference effect, and thus the comparison between this and other interference effects. In our study, we took into account each of these methodological aspects, discussed in more detail below, in order to study the interference effect across age.

In the original version of this manuscript, we focused on the specific measure of interference to disentangle whether the ratio of congruent and incongruent (ratio scores) or the difference between these conditions (absolute scores) was a more appropriate measure of the general interference effect. As reported, we did not find evidence to suggest that measuring the interference effect using absolute scores is better than the ratio scores of the interference effect or vice versa. To simplify analysis in subsequent sections, we chose the absolute scores to measure the interference effects because the data were distributed marginally more normally than the ratio scores. This is not to imply that the normal distribution is the correct way to model these data, rather this was a somewhat arbitrary choice to simplify the analysis. Furthermore, we did not model individual RTs, but rather the across-participant distribution of mean RTs and IES per participant and condition. Without further exploration or modelling, both methods could reasonably be used to measure the interference effect.

We examined whether response interference was greater in stimulus- compared to response-balanced conditions. Unexpectedly, we did not find a significant difference in interference effects between these conditions. Although the difference in response interference effects between stimulus- and response-balanced conditions was in the predicted direction, our manipulation of the proportion of response-congruent trials was not successful. Schmidt and Besner (2008) reported a 27 ms and 5% advantage in their stimulus-balanced (609 ms and 32%) compared to their response-balanced condition (636 ms and 37%) when reaction times and percentage of errors were analysed separately. To facilitate the comparison between studies and to interpret these results, we re-analysed the percentage of correct responses and reaction times separately. We found that interference effects were comparable between stimulus- and response-balanced conditions for percentage correct responses, but for reaction times the perceptual interference effect was 28 ms greater in stimulus- (41 ms) than response-balanced conditions (13 ms). Our results therefore match well the 27 ms differences in the previous study (Schmidt and Besner 2008). We suppose that in our study, facilitation in the stimulus-balanced condition may have been obscured in part by accuracy ceiling effects in each age group.

Taking into account the above methodological aspects, the main question was to understand how children, younger and older adults resolve perceptual, response and general interference. In our study, the interference effects significantly decreased with age: younger people showed more difficulties compared to older people when they had to ignore irrelevant information. Specifically, children experienced more perceptual, response and general interference compared to young adults, who showed more interference compared to older adults. Our findings are consistent with previous studies (Jongen and Jonkman 2008; Cragg 2016) in which a significant interaction between interference at the stimulus level and age was reported. The perceptual interference effect decreased with age; specifically, children showed longer reaction times (Cragg 2016) in comparison to young adults. In the same direction for the response interference effect, Jongen and Jonkman (2008) reported that children showed more errors in comparison to young adults when they had to solve the interference originating from conflicting responses. Our results, however, seem to be inconsistent with other findings (Killikelly and Szucs 2013; Cragg 2016; Hirst et al. 2019). Cragg (2016) reported that children and young adults showed comparable performance in the presence of interfering responses. Both Killikelly and Szucs (2013) and Hirst et al. (2019) did not find a significant effect of age with either perceptual or response interference effects in terms of both accuracy and reaction times. We could not compare the sample recruited in Killikelly and colleagues’ study, because the authors tested adolescents, younger and middle-age adults and our sample was not comparable in terms of age. In order to better compare the studies, we reanalysed the original data from two similar previous studies (Cragg 2016; Hirst et al. 2019), adjusting for the measure of interference effect (absolute scores). In our study, children showed significant perceptual and response interference effects. These findings are in line with Cragg’s results in which children of the same age showed significant perceptual and response interference. Regarding the middle group, we found that young adults showed interference at each level, also in this case our findings are similar to Cragg’s findings which showed a significant response interference in the young adults group. Finally, our data showed that older adults are not significantly vulnerable to the influence of interfering stimuli. These data do not therefore agree with the finding of Hirst and colleagues in which older adults showed a significant perceptual interference effect.

At this point, we speculated on reasons why children were more impacted from both stimulus and response interference, whereas older adults were most protected. Our findings disagree with a U-shaped lifespan developmental function of stimulus and response conflict cost. Li et al. (2009) reported a rapid decrease of interference cost from early childhood to early adulthood, which then remained stable in adulthood, and gradually increased from early to late old age. In a different study, Ratcliff et al. (2010, 2012) reported that the slowdown of the reaction times recorded at both ends of the lifespan curve had different sources. Using drift diffusion modelling, the authors analysed decision and non-decision components in traditional measures of processing speed. Children and older adults waited for more evidence before making a response and spent more time for stimulus encoding and response execution. In other words, children and older adults were both slow in non-decision components, however older adults were faster in their ability to extract information from stimuli. Despite global similarities in reaction time distributions between children and older adults, the underlying cognitive mechanisms may differ. Further studies will be required to investigate components of decision-making process underpinning the interference control.

After a reviewer’s suggestion, we log-transformed the interference effects to deal with different levels of variability in the three age groups. The results are presented in supplementary material—Sect. 7. We found comparable perceptual, response and general interference effect between stimulus and response balanced conditions. We did not find any significant effect of age for the perceptual interference effect. The response and general interference effects decreased with age. Looking at the data, we found that children and young adults showed comparable interference effects which significantly decreased in comparison to older adults.

In supplementary material—Sect. 8, we have re-analysed the data using ratio scores. We found that perceptual, response and general interference effects were comparable between the stimulus and response balanced conditions. In addition, we found a significant effect of age for the response and general interference, but we did not find the same influence for the perceptual interference effect. Looking at the data, children and young adults showed comparable interference, and both groups showed more interference compared to older adults. These findings are different from what we found when using the absolute scores. The non-linear nature of the transformation required to produce both ratio scores and IES, in the presence of baseline differences between groups, may impact the interpretation of interference scores. This effect may lead to discrepancies in the directions of between-group differences in absolute scores and ratio scores. We would encourage authors to explore the consequences of different kinds of analysis (while adhering to best-practice and to a priori or pre-registered analyses), perhaps by conducting a multiverse analysis of interference effects across the lifespan.

In our study there are some limitations. First, there were differences in experimental setting between the different age groups, most importantly the number of practice blocks and the number of trials. In order to control for this aspect, we reanalysed the data using the same number of trials (64) for each age group, and we still found the same effect of age on the interference effects. Second, some participants were tested during public outreach events and others were tested under stricter laboratory situations, using different computer screens and keyboards. While these differences should affect all three experimental conditions similarly (C, SI, SRI), it is possible that the within-participant effect-sizes depended partly on these experimental factors. Future studies should aim to minimise experimental differences between age groups, wherever possible. Third, we did not perform an a priori power analysis to justify the sizes of either group. Considering the effect sizes of perceptual interference, as presented in Table 3, future studies aiming to replicate this particular finding would need to recruit 64 children, 4 young adults, and 304 older adults to have an 80% chance of finding a one-tailed perceptual interference that is significantly above zero, for each group separately. These estimated sample sizes reflect the effect-sizes that we found in our work. Finally, it remains to be determined in future studies whether the version of the Flanker task that we studied will be useful as a cognitive marker of individual differences. In one study, Hedge et al. (2018) showed a relatively low test–retest reliability (between 0.40 and 0.57) of the arrow-based Flanker task when testing homogeneous groups of mostly young adult psychology undergraduate students from a university population. Our study recruited from three diverse age groups, spanning a very large portion of the lifespan, and we did not recruit a majority of psychology undergraduate students into our younger adult sample. It is important for future research to study the test–retest reliability of the strong effects of participant age that we have found.

## Conclusion

We found that people of all ages experienced significant interference when they had to ignore irrelevant information originating from interfering stimuli, from interfering responses and from both at the same time. We also found that interference effects decreased significantly with age. However, comparing the data across three similar reports, the effect-sizes varied substantially both across studies and age groups. Perceptual interference effects were, overall, quite weak, while response and general interference effects were much stronger. Meta-analytic methods, modelling, or large-scale studies with identical methods across age groups may now be required to answer the question whether perceptual and response interference changes significantly across the lifespan.

### Supplementary Information

Below is the link to the electronic supplementary material.

Supplementary file1 (DOCX 168 KB)

## Data Availability

Code and raw data are freely available on OSF at https://osf.io/sx3je/.
