# Supplementary material for: Flanker interference at both stimulus and response levels decreases with age
Source: Exp Brain Res. 2024 Feb 2;242(3):757–67. doi: 10.1007/s00221-023-06773-9 (PMC10894761; doi:10.1007/s00221-023-06773-9)
Supplement: Supplementary file 1 — Supplementary file1 (DOCX 168 KB) [file 221_2023_6773_MOESM1_ESM.docx]

**Supplementary material**

**Article title:** Flanker interference at both stimulus and response levels decreases with age

**Journal name**: Experimental Brain Research

**Authors’ information**

**Authors’ names:** Nunzia Valentina Di Chiaro^1^, Nicholas Paul Holmes^2^

**Affiliations**: 1. School of Psychology, University of Nottingham, University Park, Nottingham, United Kingdom. 2. School of Sport, Exercise and Rehabilitation Sciences, University of Birmingham, Birmingham, United Kingdom.

**Corresponding author:**

Nunzia Valentina Di Chiaro

Mail: valedichiaro@yahoo.it

**Supplementary material – section 1**


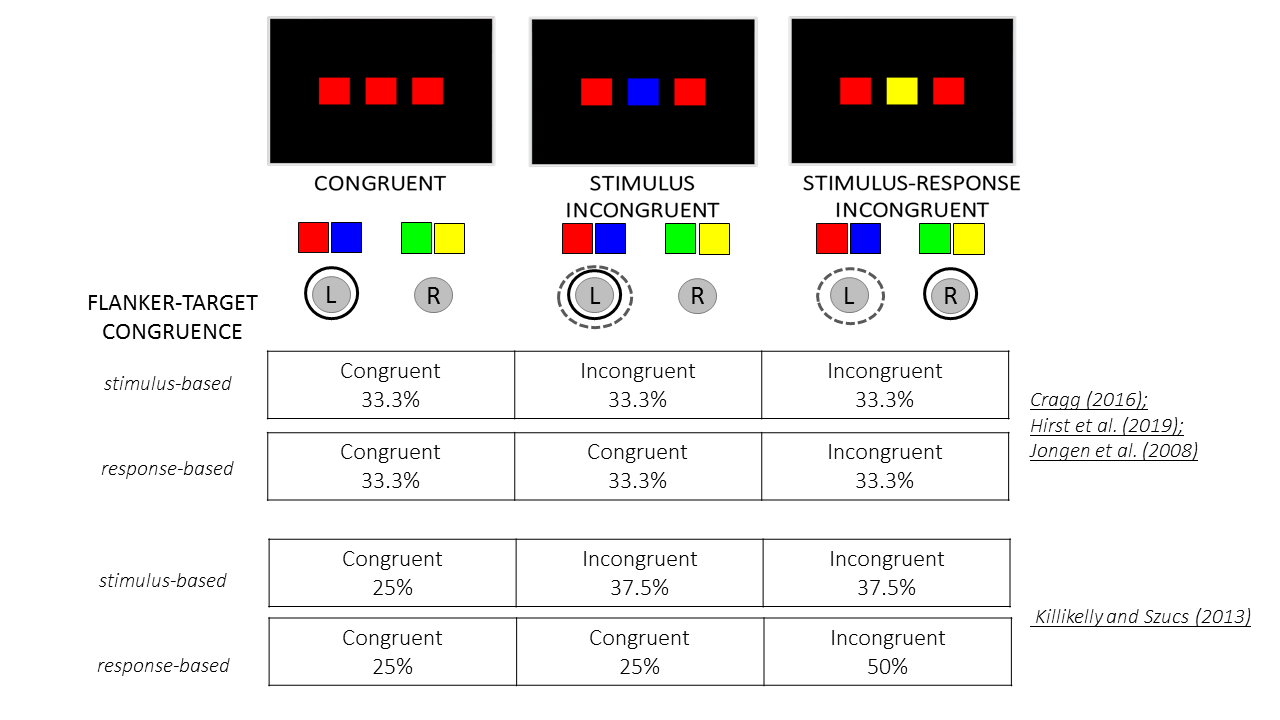


***Fig. S1*** *We used our experimental task as a schematic illustration to explain, in previous studies, how the proportion of trials for congruent, stimulus-incongruent and stimulus-response incongruent condition influences differently the flanker-target congruence when it was stimulus- and response-based. For each condition, correct response has been circled with a solid black line and the incorrect response in a broken grey line. L: left response button, R: right response button*

**Supplementary material – section 2**

**Correlation between MOCA score and perceptual, response and general interference effects**

In older adults, we did not find any significant association between the MOCA score (M=25.97, SD=2.50) and the perceptual (M=6.84 ms, SD=47.9, r(28)=.136, p=.472), response (M=30.3 ms, SD=65.3, r(28)=.048 p=.799) and general interference effect (M=37.2 ms, SD=47.3, r(28)=.214, p=.256).

**Correlation between accuracy and RTs**

Inverse efficiency scores (IES) are recommended as measure of performance when the number of errors is less than 10% and when the accuracy and speed of performance are correlated. In our study, a ceiling effect was shown for the accuracy, specifically all age groups reached at least 90% correct responses (older adults: M=97.2%, SD=4.30; children M=94.1%, SD=6.14 and young adults (M=92.6%, SD=4.92). A logistic transformation of the proportion of percentage of correct responses between blocks was performed to address the skewed distribution of the accuracy. We analysed in the total sample the association between the accuracy and the RTs using the Pearson’s correlation. We found that better accuracy scores were associated with better RTs (M=3.36, SD=1.15; M=737, SD=221 respectively; r(134)=-.211 p=.014).

**Supplementary material – section 3**

**Measures of interference effect**

The ratio scores and absolute scores of the general interference effect were significantly correlated (r(134)=.865, p<.001). Greater interference effect measured using ratio scores correlated with greater interference effect measured using absolutes scores. A bootstrap resampling technique with 10000 iterations was used to investigate whether ratio or absolute scores were better – in terms of their distributions – as a parametric measure of the interference effect.

This analysis showed that resampled ratio and absolute scores were similarly normally distributed, although a Q-Q plot of the original raw data revealed a larger deviation from normality at the upper tail for general interference effects measured using ratio scores in comparison to absolute scores. Specifically, RT distribution across participants was slightly positively skewed when the interference effect was measured using ratio scores. Overall, there was no strong statistical reason to conclude that one measure was better than the other, and both relative and absolute scores may be good measures of the interference effect. After fitting our models, the residuals were reasonably normal, such that the normal distribution was appropriate for our study. Only for the reason of simplifying the number of statistical tests in the subsequent sections, we arbitrarily chose the absolute scores based on their marginally more normal distributions.

**Supplementary material – section 4**

| **Study** | **Ages**  **(years)** | **N** | **Perceptual interference** | | | | **Response interference** | | | | **General interference** | | | |
| --- | --- | --- | --- | --- | --- | --- | --- | --- | --- | --- | --- | --- | --- | --- |
|  |  |  | **IES** | **(SD)** | **SE** | ***d*** | **IES** | **(SD)** | **SE** | ***d*** | **IES** | **SD** | **SE** | ***d*** |
| Our study | 6-14 | 81 | 40.8 | (129) | 14.3 | 0.32 | 118 | (127) | 14.1 | 0.93 | 158 | (196) | 21.8 | 0.81 |
|  | 20-43 | 24 | 32.7 | (19.9) | 4.1 | 1.64 | 71.1 | (66.5) | 13.6 | 1.07 | 104 | (67.7) | 13.8 | 1.54 |
|  | 60-84 | 31 | 6.84 | (47.9) | 8.6 | 0.14 | 30.3 | (65.3) | 11.7 | 0.46 | 37.2 | (47.3) | 8.5 | 0.79 |
| Cragg (2016) | 7-10 | 76 | 176 | (177) | 20.3 | 0.99 | 259 | (384) | 44.1 | 0.67 | 435 | (468) | 53.7 | 0.93 |
|  | Adults | 38 | -2.76 | (45.4) | 7.4 | -0.06 | 92.4 | (62.8) | 10.2 | 1.47 | 89.6 | (58.5) | 9.5 | 1.53 |
| Hirst et al (2019) | 6-11 | 49 | 41.1 | (227) | 32.4 | 0.18 | 80.1 | (305) | 43.6 | 0.26 | 121 | (289) | 41.3 | 0.42 |
|  | 18-25 | 33 | 14.0 | (50.9) | 8.9 | 0.28 | 72.5 | (118) | 20.5 | 0.61 | 86.6 | (107) | 18.6 | 0.81 |
|  | 61-85 | 38 | 69.4 | (89.1) | 14.5 | 0.78 | 194 | (622) | 101 | 0.31 | 263 | (591) | 95.9 | 0.45 |

***Table S1*** *Comparing the current data with Cragg (2016) and Hirst et al. (2019). Inverse efficiency scores (IES), standard deviation (SD) and Cohen’s d calculated for each age group. In Cragg, we calculated pooled IES and SD to combine 7 and 10 years olds in a single group. N: number of participants.*

**Supplementary material – section 5**

**Results after excluding one outlier**

Following a reviewer's request, we re-analysed the data excluding one outlier belonging to the children group. The ratio and absolute scores of the general interference effect were significantly correlated (r(133)=.883, p<.001). Resampled ratio and absolute scores were similarly normally distributed, again the Q-Q plot revealed a larger deviation from normality at the upper tail for general interference effect measured using ratio scores in comparison to absolute scores. There was no significant effect of condition (s*timulus-balanced versus response-balanced*) for perceptual, response and general interference (F(1,133)=2.36, p=.127, ηp²=.0174, MSE=12599; F(1,133)=0.036, p=.849, ηp²=.000271, MSE=24701; F(1,133)=1.72 p=.192, ηp²=.0128, MSE=23903 respectively) and no significant interaction between condition and age for perceptual, response and general interference effect (F(1,133)=0.930, p=.337, ηp²=.00694, MSE=12599; F(1,133)=0.016, p=.898, ηp²=.00012, MSE=24701; F(1,133)=0.325, p=.570, ηp²=.00244, MSE=23903, respectively) **(Table S2).** We did not find any significant effect of age for the perceptual interference effect (F(1,133)=3.89, p=.051, ηp²=.0284, MSE=29384), whereas a significant effect of age was found for the response and general interference effects (F(1,133)=16.0, p<.001, ηp²=.107, MSE=8858; F(1,133)=23.2, p<.001, ηp²=.149, MSE=12931 respectively) **(Table S3**). Younger people experienced more response interference than older people. Regarding the general interference, children and young adults showed comparable interference, and both groups experienced greater interference compared to older adults.

|  | **Stimulus-balanced**  ***(75% response-congruency)*** | | | **Response-balanced**  ***(50% response-congruency)*** | | |
| --- | --- | --- | --- | --- | --- | --- |
|  | **M** | **SD** | ***d*** | **M** | **SD** | ***d*** |
| Perceptual interference | 41.3 | 117 | 0.353 | 12.6 | 120 | 0.105 |
| Response interference | 95.1 | 164 | 0.580 | 74.7 | 132 | 0.566 |
| General interference | 136 | 161 | 0.845 | 87.3 | 168 | 0.520 |

***Table S2.*** *Mean (M), standard deviation (SD), effect size (Cohen’s d) of interference effects (IES, ms) in stimulus and response-balanced conditions after excluding one outlier.*

|  | **Children**  **(N=80)** | | | | | | **Young adults**  **(N=24)** | | | | | | **Older adults**  **(N=31)** | | | | | |
| --- | --- | --- | --- | --- | --- | --- | --- | --- | --- | --- | --- | --- | --- | --- | --- | --- | --- | --- |
|  | **M** | | **SD** | | ***d*** | | **M** | | **SD** | | ***d*** | | **M** | | **SD** | | ***d*** | |
| Perceptual | 33.0 | | 109 | | 0.303 | | 32.7 | | 20.0 | | 1.635 | | 6.84 | | 47.9 | | 0.143 | |
| Response | 110 | | 109 | | 1.01 | | 71.1 | | 66.5 | | 1.069 | | 30.3 | | 65.3 | | 0.464 | |
| General | 143 | | 142 | | 1.01 | | 104 | | 67.7 | | 1.536 | | 37.2 | | 47.3 | | 0.786 | |
|  |  | |  | |  | |  | |  | |  | |  | |  | |  | |
|  | **Children vs Young adults** | | | | | | **Children vs Older adults** | | | | | | **Young adults vs Older** | | | | | |
|  | **t** | **df** | | ***d*** | | **p** | **t** | **df** | | ***d*** | | **p** | **t** | **df** | | ***d*** | | **p** |
| Perceptual | 0.021 | 93.6 | | 0.002 | | .983 | 1.75 | 107 | | 0.166 | | .082 | 2.72 | 42.2 | | 0.367 | | .009 |
| Response | 2.14 | 63.2 | | 0.210 | | .036 | 4.72 | 90.0 | | 0.448 | | <.001 | 2.27 | 49.1 | | 0.306 | | .028 |
| General | 1.87 | 82.2 | | 0.183 | | .065 | 5.89 | 107 | | 0.559 | | <.001 | 4.11 | 39.3 | | 0.554 | | <.001 |

***Table S3.*** *Interference effects (IES, ms) in age groups after excluding one outlier. Mean (M), standard deviation (SD), effect size (Cohen’s d), T-test (t), degree of freedom (df).*

**Supplementary material – section 6**

**Results using the same number of trials across groups**

We did not find any significant interaction between condition and age for perceptual, response and general interference effect (F(1,134)=0.118, p=.732, ηp²=.00088, MSE=16817; F(1,134)=0.284, p=.595, ηp²=.00212 MSE=27466; F(1,134)=0.633, p=.428, ηp²=.00470, MSE=27863, respectively) **(Table S4)**. Children experienced more perceptual and response interference than older adults, whereas young adults showed comparable interference effects with children and older adults. Regarding the general interference, children and young adults showed comparable interference, and both groups experienced greater interference compared to older adults **(Table S5).**

|  | **Stimulus-balanced**  ***(75% response-congruency)*** | | | **Response-balanced**  ***(50% response-congruency)*** | | |
| --- | --- | --- | --- | --- | --- | --- |
|  | **M** | **SD** | ***d*** | **M** | **SD** | ***d*** |
| Perceptual interference | 41.0 | 125 | 0.328 | 14.0 | 155 | 0.090 |
| Response interference | 96.0 | 181 | 0.530 | 86.2 | 152 | 0.567 |
| General interference | 137 | 184 | 0.745 | 100 | 224 | 0.446 |

***Table S4.*** *Mean (M), standard deviation (SD), effect size (Cohen’s d) of interference effects (IES, ms) in stimulus and response balanced conditions using the same number of trials across groups.*

|  | **Children**  **(N=81)** | | | | | | **Young adults**  **(N=24)** | | | | | | **Older adults**  **(N=31)** | | | | | |
| --- | --- | --- | --- | --- | --- | --- | --- | --- | --- | --- | --- | --- | --- | --- | --- | --- | --- | --- |
|  | **M** | | **SD** | | ***d*** | | **M** | | **SD** | | ***d*** | | **M** | | **SD** | | ***d*** | |
| Perceptual | 40.8 | | 129 | | 0.316 | | 19.4 | | 55.9 | | 0.347 | | -0.919 | | 58.6 | | -0.016 | |
| Response | 118 | | 127 | | 0.929 | | 71.0 | | 101 | | 0.703 | | 36.4 | | 85.5 | | 0.426 | |
| General | 158 | | 196 | | 0.806 | | 90.4 | | 96.3 | | 0.939 | | 35.5 | | 67.0 | | 0.530 | |
|  |  | |  | |  | |  | |  | |  | |  | |  | |  | |
|  | **Children vs Young adults** | | | | | | **Children vs Older adults** | | | | | | **Young adults vs Older** | | | | | |
|  | **T** | **df** | | ***d*** | | **p** | **t** | **df** | | ***d*** | | **p** | **t** | **df** | | ***d*** | | **p** |
| Perceptual | 1.17 | 89 | | 0.114 | | .246 | 2.35 | 107 | | 0.222 | | .021 | 1.30 | 53 | | 0.175 | | .199 |
| Response | 1.65 | 103 | | 0.161 | | .103 | 3.28 | 110 | | 0.310 | | .001 | 1.38 | 53 | | 0.186 | | .175 |
| General | 1.64 | 103 | | 0.160 | | .105 | 4.93 | 109 | | 0.466 | | <.001 | 2.49 | 53 | | 0.336 | | .016 |

***Table S5.*** *Interference effects (IES, ms) in age groups using the same number of trials. Mean (M), standard deviation (SD), effect size (Cohen’s d),* *T-test (t), degree of freedom (df).*

**Supplementary material – Section 7**

**Results after log-transformation of interference effects**

After a reviewer’s request, we re-analysed the data using log 10 transformation of interference effects. Resampled ratio and absolute scores were similarly normally distributed and the Q-Q plot did not show distribution difference for general interference effect measured using ratio scores in comparison to absolute scores. We found no significant effect of condition (s*timulus-balanced versus response-balanced*) for perceptual, response and general interference (F(1,134)=0.842, p=.361, ηp²=.00624, MSE=0.063; F(1,134)=1.40, p=.240, ηp²=.01034, MSE=0.165; F(1,134)=0.426 p=.515, ηp²=.00317, MSE=0.145 respectively) and no significant interaction between condition and age for perceptual, response and general interference effect (F(1,134)=1.06, p=.306, ηp²=.00785, MSE=0.063; F(1,134)=0.709, p=.401, ηp²=.00526, MSE=0.165; F(1,134)=0.047, p=.828, ηp²=.000351, MSE=0.145, respectively) **(Table S6).**

We did not find a significant effect of age for the perceptual interference effect (F(1,134)=2.54, p=.113, ηp²= .0186, MSE=0.048). For response and general interference effect, a significant effect of age was found (F(1,134)=4.23, p=.042, ηp²=.0306, MSE=0.092; F(1,134)=8.34, p=.005, ηp²=.0586, MSE=0.113 respectively) **(Table S7).** In summary, the response and general interference effects decreased significantly with age, children and young adults showed comparable interference, and both groups experienced greater interference compared to older adults.

|  | **Stimulus-balanced**  ***(75% response-congruency)*** | | | **Response-balanced**  ***(50% response-congruency)*** | | |
| --- | --- | --- | --- | --- | --- | --- |
|  | **M** | **SD** | ***d*** | **M** | **SD** | ***d*** |
| Perceptual interference | 0.0196 | 0.250 | 0.0784 | 0.0128 | 0.311 | 0.0411 |
| Response interference | 0.209 | 0.470 | 0.445 | 0.146 | 0.362 | 0.403 |
| General interference | 0.228 | 0.402 | 0.567 | 0.159 | 0.469 | 0.339 |

***Table S6.*** *Mean (M), standard deviation (SD), effect size (Cohen’s d) of interference effects (IES, ms) in stimulus and response balanced conditions after log-transformation of interference effects.*

|  | **Children**  **(N=81)** | | | | | | **Young adults**  **(N=24)** | | | | | | **Older adults**  **(N=31)** | | | | | |
| --- | --- | --- | --- | --- | --- | --- | --- | --- | --- | --- | --- | --- | --- | --- | --- | --- | --- | --- |
|  | **M** | | **SD** | | ***d*** | | **M** | | **SD** | | ***d*** | | **M** | | **SD** | | ***d*** | |
| Perceptual | 0.0258 | | 0.265 | | 0.0974 | | 0.0430 | | 0.101 | | 0.426 | | -0.0295 | | 0.137 | | -0.215 | |
| Response | 0.206 | | 0.347 | | 0.594 | | 0.225 | | 0.267 | | 0.843 | | 0.0633 | | 0.170 | | 0.372 | |
| General | 0.232 | | 0.405 | | 0.573 | | 0.268 | | 0.262 | | 1.023 | | 0.0338 | | 0.102 | | 0.331 | |
|  |  | |  | |  | |  | |  | |  | |  | |  | |  | |
|  | **Children vs Young adults** | | | | | | **Children vs Older adults** | | | | | | **Young adults vs Older** | | | | | |
|  | **t** | **df** | | ***d*** | | **p** | **t** | **df** | | ***d*** | | **p** | **t** | **df** | | ***d*** | | **p** |
| Perceptual | 0.477 | 96.5 | | 0.047 | | .634 | 1.44 | 100 | | 0.136 | | .152 | 2.26 | 52.9 | | 0.305 | | .028 |
| Response | 0.284 | 48.3 | | 0.028 | | .777 | 2.91 | 104 | | 0.275 | | .004 | 2.60 | 36.9 | | 0.351 | | .013 |
| General | 0.517 | 58.7 | | 0.050 | | .607 | 4.08 | 101 | | 0.386 | | <.001 | 4.15 | 28.4 | | 0.560 | | <.001 |

***Table S7.*** *Interference effects (IES, ms) in age groups after log-transformation of interference effects. Mean (M), standard deviation (SD), effect size (Cohen’s d), T-test (t), degree of freedom (df).*

**Supplementary material – Section 8**

**Results** **using ratio scores**

***Stimulus-balanced versus response-balanced***

We re-analysed the data using ratio scores as measure of interference. We found comparable perceptual, response and general interference effects between the stimulus-balanced and response-balanced conditions (F(1,134)=0.605, p=.438, ηp²=.00450, MSE=.021; F(1,134)=0.123, p=.726, ηp²=.000917, MSE=.029; F(1,134)=.406 p=.525, ηp²=.003021, MSE=.037 respectively) **(Table S8)**. We did not find a significant interaction between condition and age for any interference effect (perceptual: F(1,134)=0.052, p=.819, ηp²=.000388, MSE=.021; response: F(1,134)=0.052, p=.820, ηp²=.000388 MSE=.029; general: F(1,134)=0.084, p=.772, ηp²=.000626, MSE=.037).

|  | **Stimulus-balanced**  ***(75% response-congruency)*** | | | **Response-balanced**  ***(50% response-congruency)*** | | |
| --- | --- | --- | --- | --- | --- | --- |
|  | **M** | **SD** | ***d*** | **M** | **SD** | ***d*** |
| Perceptual interference | 1.06 | 0.142 | 0.423 | 1.03 | 0.160 | 0.188 |
| Response interference | 1.15 | 0.200 | 0.750 | 1.11 | 0.162 | 0.679 |
| General interference | 1.21 | 0.222 | 0.946 | 1.14 | 0.239 | 0.586 |

***Table S8.*** *Mean (M), standard deviation (SD), effect size (Cohen’s d) of interference effects (IES, ms) in stimulus and response balanced conditions using ratio scores.*

***Perceptual, response and general interference effects across age groups***

No significant effect of age was found for the perceptual interference effect F(1,134)=2.10, p=.150, ηp²=0.0154, MSE=.013. For response and general interference, a significant effect of age was shown (F(1,134)=7.15, p=.008, ηp²=.0507, MSE=.018; F(1,134)=8.29, p=.005, ηp²=.0583, MSE=.033, respectively). Children and young adults showed comparable response and general interference, and both groups showed more response and general interference compared to older adults **(Table S9; Fig. S2**). In summary, using the ratio scores we found an effect of age only for the response and general interference. We did not find the effect of age for the perceptual interference effect.

|  | **Children**  **(N=81)** | | | | | | **Young adults**  **(N=24)** | | | | | | **Older adults**  **(N=31)** | | | | | |
| --- | --- | --- | --- | --- | --- | --- | --- | --- | --- | --- | --- | --- | --- | --- | --- | --- | --- | --- |
|  | **M** | | **SD** | | ***d*** | | **M** | | **SD** | | ***d*** | | **M** | | **SD** | | ***d*** | |
| Perceptual | 1.05 | | 0.135 | | 0.370 | | 1.08 | | 0.046 | | 1.74 | | 1.02 | | 0.071 | | 0.282 | |
| Response | 1.15 | | 0.132 | | 1.14 | | 1.16 | | 0.168 | | 0.952 | | 1.05 | | 0.090 | | 0.556 | |
| General | 1.19 | | 0.196 | | 0.969 | | 1.25 | | 0.197 | | 1.27 | | 1.07 | | 0.080 | | 0875 | |
|  |  | |  | |  | |  | |  | |  | |  | |  | |  | |
|  | **Children vs Young adults** | | | | | | **Children vs Older adults** | | | | | | **Young adults vs Older** | | | | | |
|  | **t** | **df** | | ***d*** | | **p** | **t** | **df** | | ***d*** | | **p** | **t** | **df** | | ***d*** | | **p** |
| Perceptual | 1.86 | 101 | | 0.182 | | .065 | 1.40 | 99.0 | | 0.132 | | .166 | 3.80 | 51.7 | | 0.512 | | <.001 |
| Response | 0.559 | 103 | | 0.055 | | .577 | 4.34 | 79.1 | | 0.410 | | <.001 | 3.20 | 53 | | 0.431 | | .002 |
| General | 1.41 | 103 | | 0.138 | | .162 | 4.79 | 110 | | 0.453 | | <.001 | 4.43 | 28.9 | | 0.597 | | <.001 |

***Table S9.*** *Interference effects (IES, ms) in age groups using ratio scores. Mean (M), standard deviation (SD), effect size (Cohen’s d), T-test (t), degree of freedom (df).*


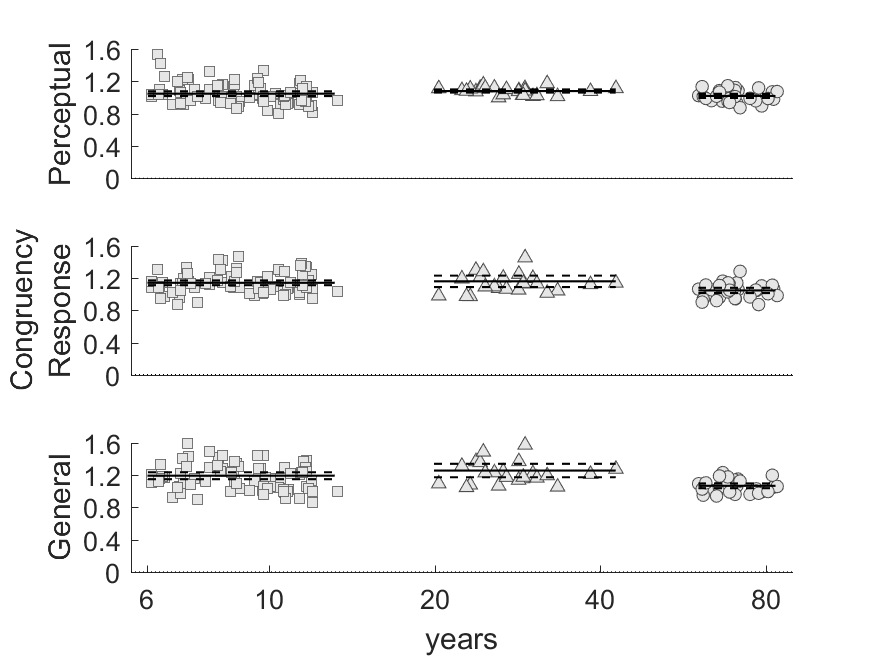


***Fig. S2*** *Perceptual, response and general interference effects are displayed across age groups using ratio scores: children (squares), young (triangles) and older adults (circles) Mean and 95% confidence interval are shown for each age group*
